# Electropolymerization of an EDOT-Quinoxaline Monomer for Green Electrochromic Thin Films and Devices

**DOI:** 10.3390/polym16060799

**Published:** 2024-03-13

**Authors:** Marco Schott, Lukas Niklaus, Silvia Janietz, Charlotte Völkel, Tatjana Egorov-Brening, Taybet Bilkay-Troni

**Affiliations:** 1 Fraunhofer Institute for Silicate Research ISC, Neunerplatz 2, 97082 Würzburg, Germany; 2 Fraunhofer Institute for Applied Polymer Research IAP, Geiselbergstr. 69, 14476 Potsdam, Germanytatjana.egorov-brening@iap.fraunhofer.de (T.E.-B.);

**Keywords:** Stille cross-coupling, electropolymerization, green electrochromic polymer, Prussian blue, electrochromic device

## Abstract

In this study, we present a 5,8-bis(3,4-ethylenedioxythiophene)quinoxaline monomer with two 4-(octyloxy)phenyl side chains (EDOTPQ) that can be electropolymerized on ITO glass in standard electrolytes containing lithium salts and propylene carbonate as solvent. The electrochemically deposited PEDOTPQ layers show very good adhesion and homogeneity on ITO. The green-colored polymer thin films exhibit promising electrochromic (EC) properties and are interesting for applications such as adaptive camouflage, as well as smart displays, labels, and sensors. Novel organic–inorganic (hybrid) EC cell configurations were realized with Prussian blue (PB) or titanium-vanadium oxide (TiVO_x_) as ion storage electrodes, showing a highly reversible and fast color change from green to light yellow.

## 1. Introduction

**Figure 1:**
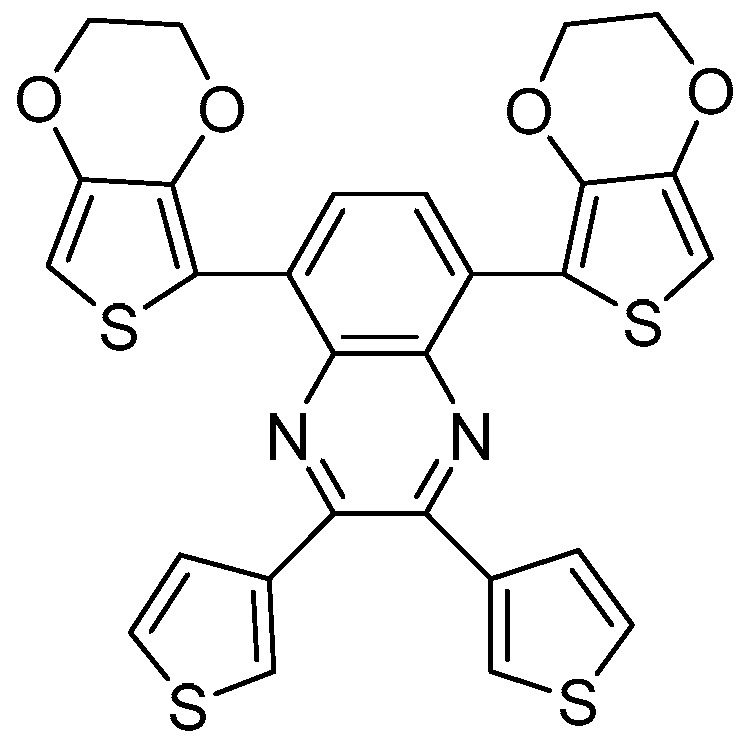
Chemical structure of the 5,8-bis(EDOT)quinoxaline monomer with two thienyl substituents at the quinoxaline ring: 5,8-bis(2,3-dihydrothieno[3,4-b][1,4]dioxin-5-yl)-2,3-di(thiophen-2-yl)quinoxaline.

Electrochromic (EC) materials are able to change their optical properties (color, absorbance/transmittance, and reflectance) by application of an electrical voltage or current. For several decades, EC materials and devices have been extensively studied for different applications [1], such as smart windows in buildings and vehicles, air cabin windows, auto-dimmable rear-view mirrors, non-emissive displays [2,3], smart labels [4], and sensors [5]. In recent years, their use in potential industrial markets has been extended to camouflage [6], including color camouflage and thermal camouflage [7]. Due to their adjustable optical properties, EC materials have attracted growing attention and research interest in this field. However, it is challenging to fulfill the harsh requirements for these applications, and further developments in the design and fabrication of novel electrochromic devices (ECDs) are necessary. Besides the well-known metal oxides, metal coordination complexes/polymers, and viologens [8], a huge variety of conjugated EC polymers can be found in the literature, showing the complete color palette [9,10,11,12,13]. Among the many outstanding conducting polymers, polypyrrole (PPy), polyaniline (PANI), poly(3,4-ethylenedioxythiophene) (PEDOT), and their derivates have been widely studied due to their invaluable properties for application in ECDs [14,15]. Conducting polymers are also used in nanocomposite electrodes to improve their EC performance, e.g., WO_3_/PPy [16]. In many studies, solution-processable EC polymers with, e.g., a blue- or red-colored neutral state, have been investigated [17,18,19,20], whereas few studies have been reported related to polymers reflecting a green to highly transmissive color change [21,22,23,24,25]. In particular, green EC materials are interesting for adaptive camouflage [26] and displays [27]. To obtain a green color in the neutral state, there must be at least two simultaneous absorption bands in the red and blue regions of the visible spectrum. In addition to the color in the neutral state of the polymer, the transmissivity in the oxidized state is of high relevance for several applications. Consequently, the material should possess two absorption bands with a specific maximum, and upon oxidation, these bands should vanish to have a completely colorless state. It has been demonstrated that the incorporation of alternating donor–acceptor units in the polymer backbone leads to a significant decrease in the band gap due to the increased double bond character in the structure [21,22]. 5,8-bis(EDOT)quinoxaline monomers with two thienyl substituents at the quinoxaline ring (see Figure 1) were electrochemically polymerized to investigate their potential use as a neutral-state green EC material. The green polymer reveals relatively high optical contrasts in the visible and NIR region of the electromagnetic spectrum paired with excellent switching properties and cycling stability [23].

**Figure 2:**
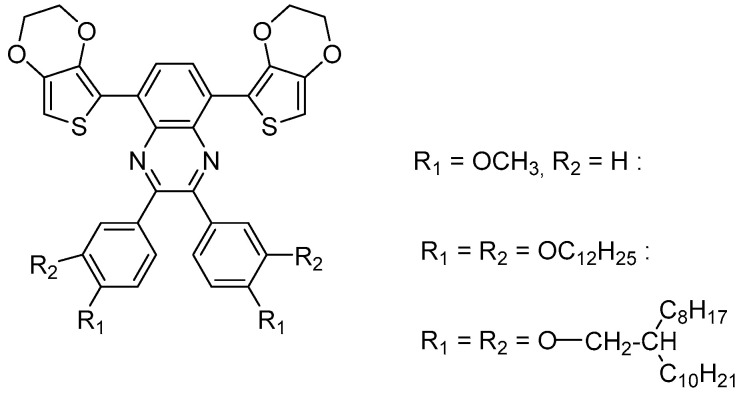
Chemical structure of the 5,8-bis(EDOT)quinoxaline monomer with two alkoxy substituents on each phenyl ring [29].

The issue of such rigid donor–acceptor–donor molecules is their low solubility in organic solvents, such as acetonitrile or propylene carbonate (PC), and thus also in standard electrolytes used for electrochemical polymerization. The electropolymerization of quinoxaline derivatives without alkyl or alkoxy substituents can only be carried out in chlorinated solvents like dichloromethane [23] or mixtures of acetonitrile and dichloromethane [28]. Specifically, the introduction of substituents with long alkyl chains can drastically improve the solubility and is of high importance for the processability and production of EC thin films. The introduction of three different alkoxy side chains (short, straight, and branched) at the two phenyl units of the quinoxaline derivative (see Figure 2) leads to polymers with improved solubility in organic solvents [29].

In particular, the derivative with two bulky alkoxy side chains and the polymer obtained by conventional chemical polymerization has excellent solubility in organic solvents, such as xylene or toluene. Therefore, the polymer could be deposited on conductive surfaces as a film from solution, e.g., by using spray or spin coating. Additionally, the EC thin films show a saturated green color in the reduced state at a low applied voltage of −1.5 V and sand colors in the oxidized state at +1.5 V. The fabrication of chameleonic textile devices has been demonstrated. The ability to reversibly change between green and sand colors is essential for adaptive camouflage [29].

Conjugated donor–acceptor copolymers based on EDOT as the donor and quinoxaline with two 4-(octyloxy)phenyl side chains as the acceptor were previously synthesized by Stille cross-coupling reaction of 5,8-dibromo-2,3-bis(4-(octyloxy)phenyl)quinoxaline with 2,5-bis(tributylstannyl)-EDOT. The polymer with low molecular weight (M_n_: 16,8 KDa) was obtained in moderate yields (65%) and is only soluble in chlorinated solvents. It was applied as a low-band-gap polymer in organic photovoltaic cells with quite low efficiencies [30].

**Figure 3:**
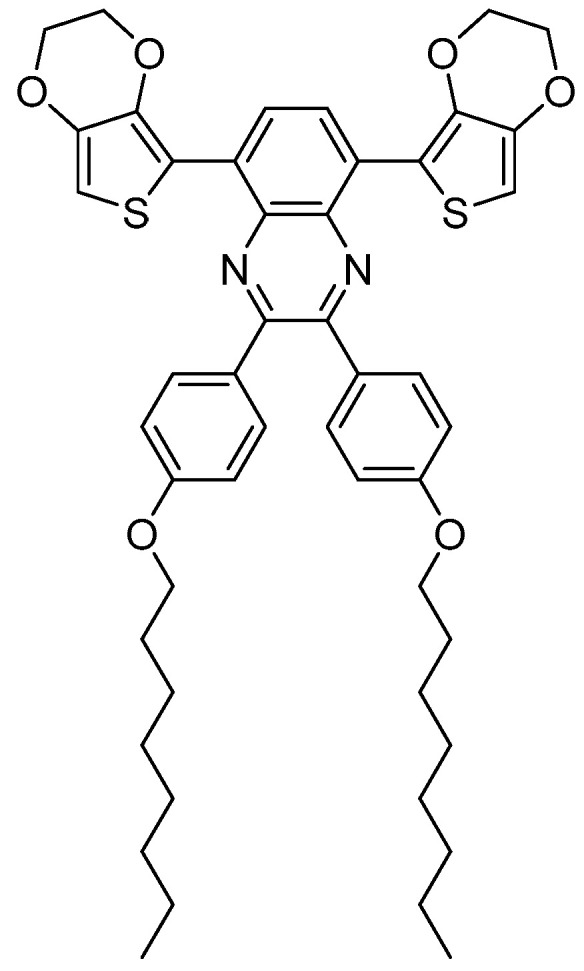
Chemical structure of the 5,8-bis(EDOT)quinoxaline monomer with two 4-(octyloxy)phenyl side chains.

In this study, we present a 5,8-bis(EDOT)quinoxaline monomer with two 4-(octyloxy)phenyl side chains (Figure 3) that were electrochemically polymerized in a standard electrolyte containing PC as the solvent.

The obtained thin films on transparent conductive glass show a vibrant green color in the neutral (reduced) state and a light yellow color in the oxidized state. The EC properties of the electrodes and full devices with two various inorganic counter electrodes are investigated in detail.

## 2. Materials and Methods

All chemicals for the monomer synthesis and the electrolyte for the electropolymerization, i.e., lithium trifluoromethanesulfonate (LiTf), PC, and poly (ethylene glycol) methyl ether acrylate (PEG-MEA) (M_n_ = 480 g/mol) were purchased from Sigma-Aldrich (Darmstadt, Germany) and used without further purification. A different electrolyte was used for the ECDs, which consists of lithium bis(trifluoromethanesulfonyl) imide (LiTFSI), PC, and diethyl carbonate (DEC) purchased from Sigma-Aldrich, and paraloid as binder from Kremer Pigmente (Aichstetten, Germany). Indium tin oxide (ITO)-coated glass sheets with a size of 5 × 5 cm^2^ (sheet resistance: approx. 15 Ω/sq) were purchased from Lumtec (New Taipeh City, Taiwan). The ITO glass substrates were thoroughly cleaned in an ultrasonic bath with acetone, in an ultrasonic bath with deionized water, and then immersed in an isopropyl alcohol bath (for 5 min each). Finally, the substrates were dried with nitrogen and heated at 60 °C for 30 min. Silicone-based double-sided adhesive tape (127 µm thick) and adhesive copper tape were purchased from 3M (Saint Paul, MN, USA).

### 2.1. Synthesis of the Monomer EDOTPQ

**Figure 4:**
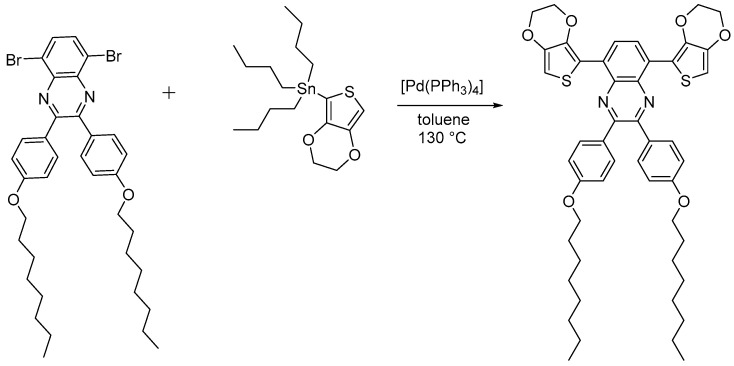
Synthesis of the 5,8-bis(EDOT)quinoxaline monomer with two 4-(octyloxy)phenyl side chains (EDOTPQ) by Stille cross-coupling.

The synthesis of 5,8-dibromo-2,3-bis(4-(octyloxy)phenyl)quinoxaline [29,31] and tributyl(2,3-dihydrothieno [3,4-b][1,4]dioxin-5-yl)stannane [32] was carried out according to the previously reported methods. The 5,8-bis(EDOT)quinoxaline monomer with two 4-(octyloxy)phenyl side chains (EDOTPQ) was synthesized by Stille cross-coupling (Figure 4).

In a 50 mL two-neck flask under argon atmosphere fitted with a reflux condenser and septum, 5,8-dibromo-2,3-bis(4-(octyloxy)phenyl)quinoxaline (1.22 g, 1.75 mmol, 1 eq.), tributyl(2,3-dihydrothieno[3,4-b][1,4]dioxin-5-yl)stannane (1.89 g, 4.38 mmol, 2.5 eq.), and tetrakis(triphenylphosphine)palladium(0) (283 mg, 0.25 mmol) were dissolved in 13.6 mL of dry toluene. The reaction mixture was heated to reflux and stirred for 15 h. After the reaction was finished, the mixture was cooled to 22 °C and added to 1 M sodium carbonate solution. The aqueous phase was extracted with dichloromethane. The organic phase was dried over anhydrous magnesium sulfate, filtered, and concentrated under reduced pressure. Purification was carried out by column chromatography in heptane/ethyl acetate. An orange solid was obtained (1.17 g, 1.43 mmol, 82% yield), R_f_: 0.33 in heptane/ethyl acetate 3:1 (*v*/*v*), mp: 162 °C. Anal. calcd for C_18_H_54_N_2_O_6_S_2_: C, 70.39; H, 6.65; N, 3.42; S; 7.83; found: C, 70.10; H, 6.66; N, 3.36; S, 7.63. ^1^H NMR (500 MHz, CDCl_3_): δ (ppm) 8.58 (s, 2H), 7.72 (d, J = 8.4 Hz, 4H), 6.89 (d, J = 8.4 Hz, 4H), 6.54 (s, 2H), 4.38 (d, J = 4.4 Hz, 4H), 4.31 (d, J = 3.6 Hz, 4H), 4.00 (t, J = 6.6 Hz, 4H), 1.80 (p, J = 6.8 Hz, 4H), 1.47 (p, J = 7.1 Hz, 4H), 1.39–1.24 (m, 16H), 0.90 (t, J = 6.7, Hz, 6H). ^13^C NMR (500 MHz, CDCl_3_): δ (ppm) 159.62, 150.14, 141.16, 140.00, 136.68, 131.76, 130.92, 128.27, 127.40, 113.95, 102.75, 67.84, 64.75, 64.17, 31.64, 29.09, 25.89, 22.48, 13.93.

### 2.2. Electropolymerization of EDOTPQ

**Figure A2:**
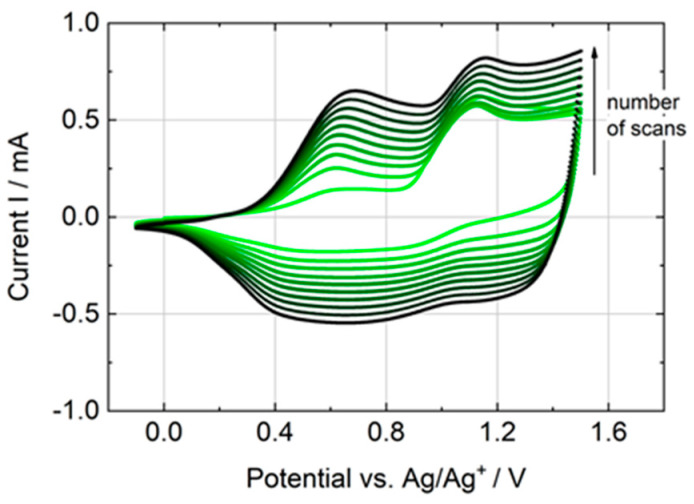
Cyclic voltammograms (scan rate: 100 mV·s^−1^) recorded during the electropolymerization of EDOTPQ on ITO glass. Ag wire as counter and pseudo-reference electrode. Electrolyte: 0.1 M TBABF_4_/acetonitrile.

The electropolymerization of the EDOTPQ monomer was carried out in solution (14 mM) by cyclic voltammetry in a three-electrode setup with a Ag wire as counter and pseudo-reference electrode and 0.1 M TBABF_4_ in acetonitrile as electrolyte to demonstrate the electrochemical oxidation activity and deposition on ITO. Due to the good solubility of the monomer in acetonitrile, it was not necessary to use chlorinated solvents. With increasing the scanning cycle number, reversible redox peaks were observed at lower potentials, caused by the extended conjugation length of the electroactive polymer film [33]. After ten repeated cycles in the potential range of −0.1 V and +1.4 V vs. Ag/Ag^+^, the green-colored polymer layer was distinctly observable (see Figure A2).

**Figure 5:**
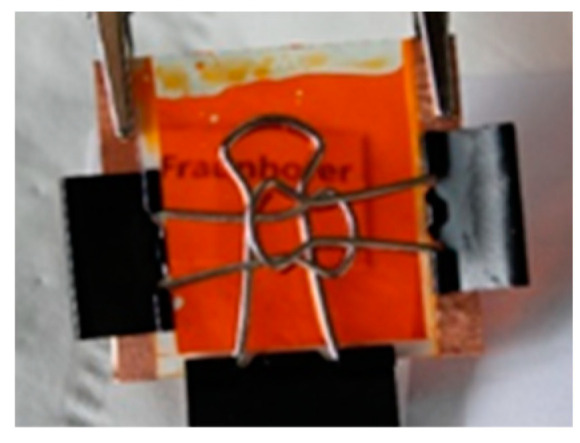
Electropolymerization of the EDOTPQ monomer dissolved in LiTf/PC/PEG-MEA as electrolyte and sandwiched between two ITO glass sheets (active area: 3.5 × 4 cm^2^).

For the electrochemical deposition of the polymer thin film on ITO glass, a modified and more simple preparation method described by Sotzing and coworkers was used [34,35]. The electrolyte for the electropolymerization was composed of 0.46 g LiTf, 1.37 g PC, 3.19 g PEG-MEA, and 0.05 g (0.06 mmol, 0.99 wt%) of the monomer EDOTPQ. The electrolyte was stirred for 1 h at room temperature and 10 min at 50 °C to obtain a homogeneous solution. Three layers of a silicone-based double-sided adhesive tape (127 µm thick) were stuck on one ITO glass (5 × 5 cm^2^) to obtain an active area of 3.5 × 4 cm^2^ and to avoid electrolyte leakage. Another piece of ITO glass was placed on top with an offset of 5 mm and fixed with binder clips. To ensure a good electrical contact, both ITO glass sheets were covered on one side with 5 mm Cu tape. The electrolyte solution (0.4 mL) was filled between the two ITO glass sheets (Figure 5).

For the electropolymerization, a two-electrode setup without a reference electrode was used. A constant voltage of 3 V was applied for 30 s and, finally, the voltage was switched to −0.5 V for 10 s, converting the monomer in the liquid state to form the polymer film on the ITO glass used as the anode. The second ITO glass (cathode) was removed and the resulting polymer film was rinsed with isopropyl alcohol to wash out the residual monomers and then dried in vacuo at 40 °C over night. The electropolymerization was performed with a PGSTAT204 potentiostat from Metrohm (Utrecht, The Netherlands).

### 2.3. Preparation of the Counter Electrodes

The synthesis of the aqueous Prussian blue (PB, Fe_4_[Fe(CN)_6_]_3_) nanoparticle ink and the preparation of PB thin films on ITO-coated polyethylene terephthalate PET from Eastman Chemical Company (Kingsport, TN, USA, Flexvue^TM^ OC50, sheet resistance: approx. 50 Ω/sq) is described elsewhere [17,36].

The sputter-deposited titanium-vanadium oxide (TiVO_x_) electrodes [37] on fluorine-doped tin oxide (FTO) coated glass (TEC^TM^ 15 from Pilkington, sheet resistance: approx. 15 Ω/sq) were provided by EControl-Glas GmbH & Co. KG (Plauen, Germany).

### 2.4. Fabrication of the Electrochromic Devices

The electrochromic devices (ECDs) with two different cell configurations [substrate/transparent conductive oxide (TCO)/PEDOTPQ/electrolyte/PB or TiVO_x_/TCO/substrate] were fabricated with an active (switchable) area of 3.5 × 4 cm^2^. The ECDs were assembled with a proprietary LiTFSI-containing gel electrolyte (LiTFSI/PC/DEC/paraloid) under argon atmosphere in a glovebox (H_2_O, O_2_ < 5 ppm). An adhesive copper tape was used for electrical contact.

### 2.5. Optical, Spectroelectrochemical, and Electrochemical Characterization of the EC Electrodes and Devices

The adhesion of the green EC polymer thin film on ITO was determined using a cross-cut test according to ISO 2409 [38]. The diffuse light scattering (haze) of the electrodes was measured using a Hazegard XL-211 hazemeter (Pacific Scientific, Hollister, CA, USA) against air as the reference. The topography of the polymeric EC layers was examined with an Auriga 60 field emission scanning electron microscope from Zeiss (Oberkochen, Germany).

UV-Vis spectra and color coordinates according to the CIE Lab color space (L* = lightness, a* = green-red, b* = blue-yellow) were recorded using an AvaSpec-3648 standard fiber optic spectrometer from Avantes (Apeldoorn, The Netherlands) equipped with a balanced deuterium-halogen light source. The visible light transmittance τ_v_ values were calculated according to DIN EN 410 [39] and Equation (1).

(1) $$\tau_{v}=\frac{\sum_{\lambda =380\;nm}^{780\;nm}D_{\lambda }T\left(\lambda \right)V(\lambda )\Delta \lambda }{\sum_{\lambda =380\;nm}^{780\;nm}D_{\lambda }T\left(\lambda \right)\Delta \lambda }$$

where λ = wavelength, D_λ_ = standard illuminant D65, T(λ) = transmittance, and V(λ) = spectral luminous efficiency of a standard observer at 10°. In situ spectroelectrochemical measurements of the EC polymer and PB thin-film electrodes were carried out under argon atmosphere (glovebox) at room temperature in a quartz cell with 1 M LiTFSI/PC as electrolyte and Li as counter and reference electrodes, respectively.

A 1470E multi-channel potentiostat from Solartron Metrodology (Leicester, UK) was used for electrochemical characterization (cyclic voltammetry, charging/discharging, and cycling stability over 1000 cycles) of the EC electrodes and the ECDs. For the half-cell measurements of the EC electrode materials (PEDOTPQ on ITO glass and PB on PET-ITO, active area: 1 × 1 cm^2^) a three-electrode setup with Li as counter and reference electrodes, and 1 M LiTFSI/PC as electrolyte under argon atmosphere (glovebox) at room temperature was used.

The spectroelectrochemical measurements of the ECDs (incl. UV-Vis and L*a*b* color coordinates of the colored and bleached states, respectively) were performed in situ during device operation. The ideal voltage range for coloring/bleaching the ECDs was determined by stepwise increasing/decreasing the cell voltage until no further transmittance changes were observed.

## 3. Results and Discussion

### 3.1. Optical Characterization of the PEDOTPQ Thin-Film Electrode

**Figure 6:**
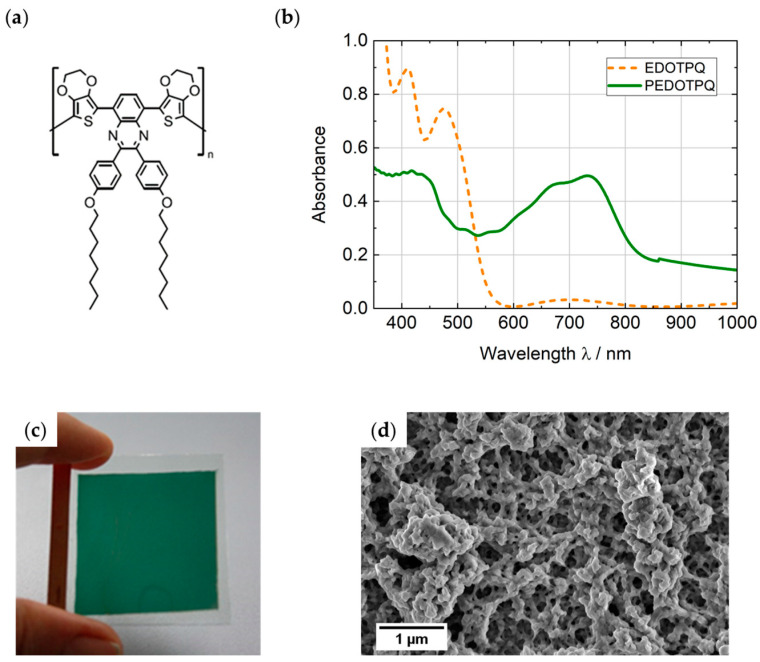
(**a**) Chemical structure of PEDOTPQ. (**b**) Absorption spectra of the monomer and polymer thin films. (**c**) Photographic image and (**d**) SEM surface image of the PEDOTPQ layer on ITO glass.

**Figure A3:**
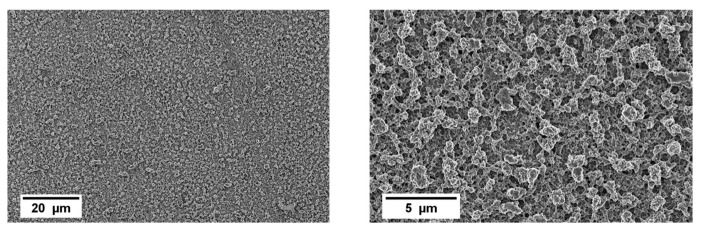
SEM images of the surface of the PEDOTPQ thin film on ITO glass with low magnification.

The electrochemically polymerized PEDOTPQ thin films (thickness: approx. 300 nm) exhibit a high homogeneity on ITO glass and intense green tint resulting from two absorption bands (λ_max_ at 418 nm and 731 nm), which were obtained by creating a donor–acceptor–donor polymer of dioxythiophene segments with intervening quinoxaline acceptors. To assess the adhesion of the polymer on ITO, a cross-cut test was performed according to ISO 2409. The cross-cut value of the PEDOTPQ thin film on ITO glass is 0, i.e., the polymer layers have a very good adhesion without any visible detachments. In addition, the diffuse light scattering (haze) was measured and the layer appears hazy, with a value of approx. 69%. Figure 6 shows the scanning electron microscope (SEM) image, depicting the surface morphology of the green EC polymer thin film on ITO glass (further SEM images with lower magnification, see Figure A3). The polymer layer is highly porous and shows no regular surface structure, which explains the high haze. However, when the PEDOTPQ electrodes are immersed with a liquid or gel electrolyte, a distinct decrease in the haze can be observed, confirming the porosity and surface roughness. Due to their high adhesion on ITO the electropolymerized PEDOTPQ films are not soluble in PC-based electrolytes.

### 3.2. Spectroelectrochemical and Electrochemical Characterization of the PEDOTPQ Electrode

**Figure 7:**
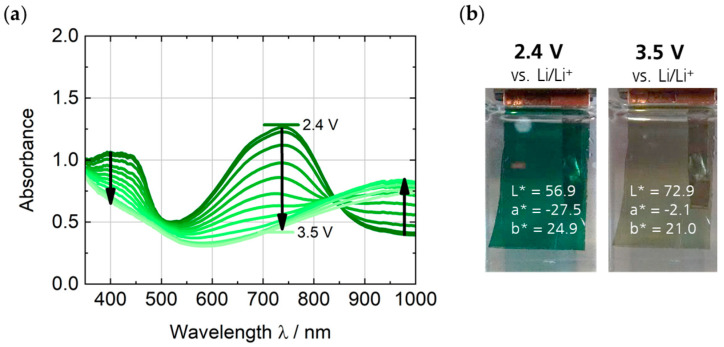
(**a**) Absorption spectra of the PEDOTPQ electrode at applied potentials in the range of 2.4 V to 3.5 V vs. Li/Li^+^. (**b**) Photographic images and L*a*b* color coordinates of the green-colored and bleached states, respectively. Counter/reference electrode: Li. Electrolyte: 1 M LiTFSI/PC.

The spectroelectrochemical characterization of the green EC polymer PEDOTPQ on ITO glass was carried out in 1 M LiTFSI/PC as electrolyte and with Li as counter and reference electrodes. The potential was increased stepwise by 0.1 V from 2.4 V up to 3.5 V vs. Li/Li^+^, and the absorption spectra were measured when no further change in absorbance or color was observed. Figure 7 shows the potential-dependent absorption spectra of the PEDOTPQ electrode and the photographic images of the green-colored (reduced) and bleached (oxidized) states. Upon switching between 2.4 V and 3.5 V vs. Li/Li^+^, the visible light transmittance τ_v_ changes from 25% to 46%. The two absorption bands with a maximum at around 400 nm and 740 nm completely disappear when switched to 3.5 V vs. Li/Li^+^ and a broad absorption band in the NIR region, with a maximum at around 970 nm appears. This results in a color change from green (L* = 56.9, a* = −27.5, b* = 24.9) to light yellow (L* = 72.9, a* = −2.1, b* = 21.0).

**Figure 8:**
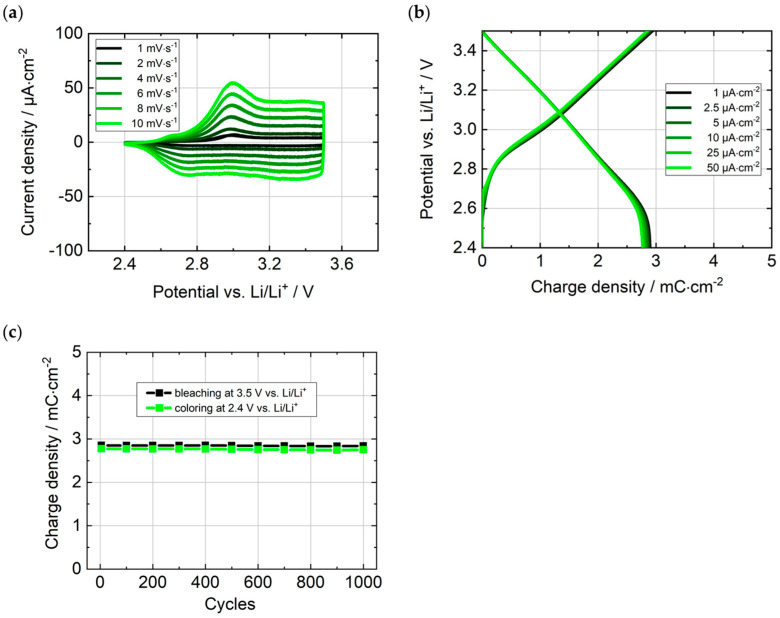
(**a**) Cyclic voltammograms at different scan rates, (**b**) galvanostatic charging/discharging at different current densities, and (**c**) 1000 switching cycles at a current density of 50 µA·cm^−2^ of the PEDOTPQ electrode. Counter/reference electrode: Li. Electrolyte: 1 M LiTFSI/PC.

The electrochemical properties of the EC electrodes were investigated in a half-cell measurement with Li as counter/reference electrode and 1 M LiTFSI/PC as electrolyte. The cyclic voltammograms in Figure 8a show broad peaks in both the oxidation (2.7 V vs. Li/Li^+^, at 10 mV s^−1^) and reduction (3.0 V vs. Li/Li^+^, at 10 mV s^−1^) of the conjugated backbone. The substantially broadened cathodic and anodic half-waves result from the hindered diffusion and insertion of the TFSI^−^ counter anions and inhomogeneities at the molecular level, which affect the redox potentials but do not cause haze [17]. In Figure 8b, the charging (bleaching) and discharging (coloring) curves of the PEDOTPQ electrode at current densities of 1 μA·cm^−2^ to 50 μA·cm^−2^ are depicted. The curves barely differ from each other when various current densities are applied due to the fast ion insertion/de-insertion favored by the porous layer structure. They also show a reversible oxidation and reduction behavior, and the maximum charge density can be determined at this almost vertical potential drop at 2.4 V vs. Li/Li^+^ in the discharge curves. At a current density of 1 μA·cm^−2^, the maximum charge density for charging and discharging is 2.94 mC·cm^−2^, resulting in a Coulombic efficiency (i.e., the percentage ratio of the discharge to the charge density) of 100%. Thus, the redox reaction of the polymer is fully reversible at low current densities. A further important figure of merit for the comparison and evaluation of EC materials is the coloration efficiency (η), which is defined as the change in optical density (ΔOD) per unit of charge inserted into or extracted from the EC layer and can be calculated according to Equation (2).

η = ΔOD/q = log(T_b_/T_c_)/q (2)

where T_b_ and T_c_ are the transmittance values in the bleached and colored states, respectively, and q is the charge density. The coloration efficiency of the PEDOTPQ thin film is 287 cm^2^·C^−1^ at λ_max_ = 736 nm. The cycling stability of PEDOTPQ was tested over 1000 switching cycles (current density: 50 μA·cm^−2^) between 2.4 V and 3.5 V vs. Li/Li^+^. The charge density of the PEDOTPQ electrode was 2.85 mC·cm^−2^ before cycling and 2.84 mC·cm^−2^ after 1000 cycles (see Figure 8c), resulting in a charge retention > 99% and indicating a high cycling stability.

### 3.3. Characterization of the Counter Electrodes

**Figure 9:**
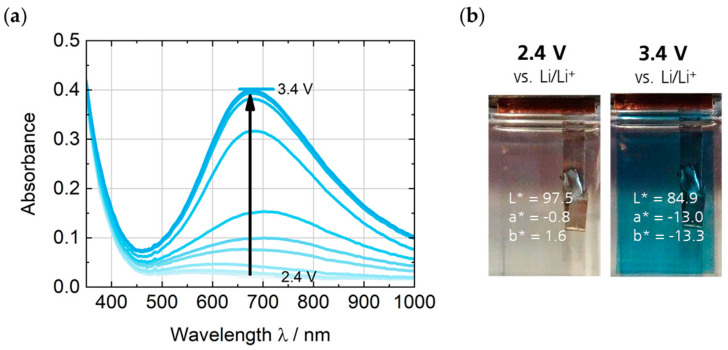
(**a**) Absorption spectra of the PB electrode at applied potentials in the range of 2.4 V to 3.4 V vs. Li/Li^+^. (**b**) Photographic images and L*a*b* color coordinates of the bleached and colored states, respectively. Counter/reference electrode: Li. Electrolyte: 1 M LiTFSI/PC.

**Figure 10:**
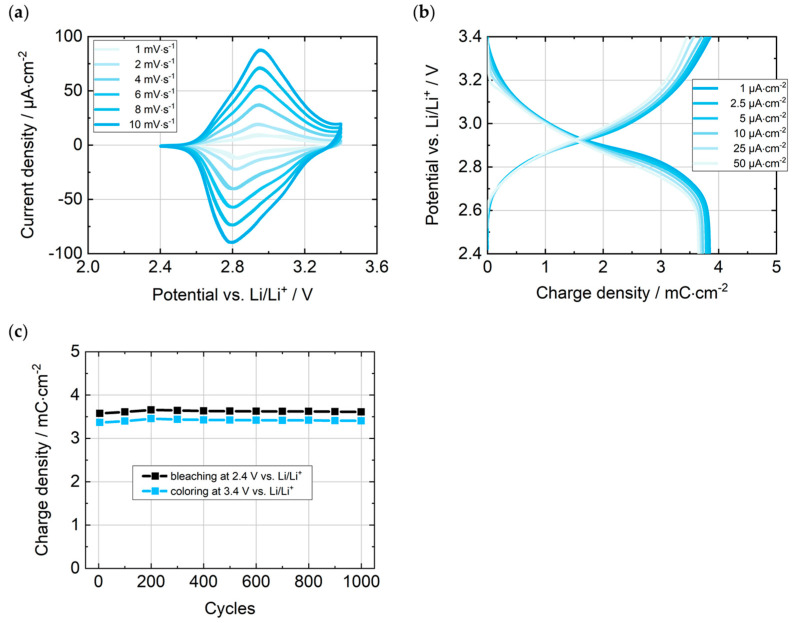
(**a**) Cyclic voltammograms at different scan rates, (**b**) galvanostatic charging/discharging at different current densities, and (**c**) 1000 switching cycles at a current density of 50 µA·cm^−2^ of the PB electrode. Counter/reference electrode: Li. Electrolyte: 1 M LiTFSI/PC.

For the fabrication of ECDs with a good EC performance, particularly high transmittance modulation, fast response, and high cycling stability, the choice of an appropriate counter electrode is important, that can be either a complementarily coloring EC electrode or a minimally color-changing (ideally optically passive) ion storage layer to ensure the charge balance. In this study, we selected PB as the anodically coloring material and TiVO_x_ for the combination with the cathodically coloring PEDOTPQ. The spectroelectrochemical (Figure 9) and electrochemical characterization (Figure 10) of the PB electrode was performed similarly to the PEDOTPQ electrode. The TiVO_x_ electrode was characterized in a previous study and serves as an effective and nearly optically passive charge storage layer in ECDs [37].

The PB thin-film electrodes switches from colorless (reduced state, Fe^2+^/^2+^) at 2.4 V vs. Li/Li^+^ to light blue (oxidized state, Fe^2+^/^3+^) at 3.4 V vs. Li/Li^+^ (Figure 10a), while the visible light transmittance changes from 94% to 64%.

From the charging/discharging (bleaching/coloring) curves in Figure 10b, the maximum charge density of the PB electrode at a current density of 1 µA·cm^−2^ was approx. 3.84 mC·cm^−2^ (at 2.4 vs. Li/Li^+^) and 3.86 mC·cm^−2^ (at 3.4 vs. Li/Li^+^). Thus, the Coulombic efficiency is approx. 100%. The charge over 1000 switching cycles between 2.4 V and 3.4 V vs. Li/Li^+^ is almost constant (3.58 mC·cm^−2^ → 3.61 mC·cm^−2^), confirming the high cycling stability of the material (Figure 10c). Due to the completely colorless reduced state and the electrochemical properties, e.g., redox potential and cycling stability, PB is an ideal candidate to be applied as a counter electrode material in ECDs containing cathodically coloring EC polymers, such as PEDOTPQ. The characteristic EC properties of the PEDOTPQ and PB electrodes are summarized in Table 1.

### 3.4. Hybrid Electrochromic Devices

**Figure 11:**
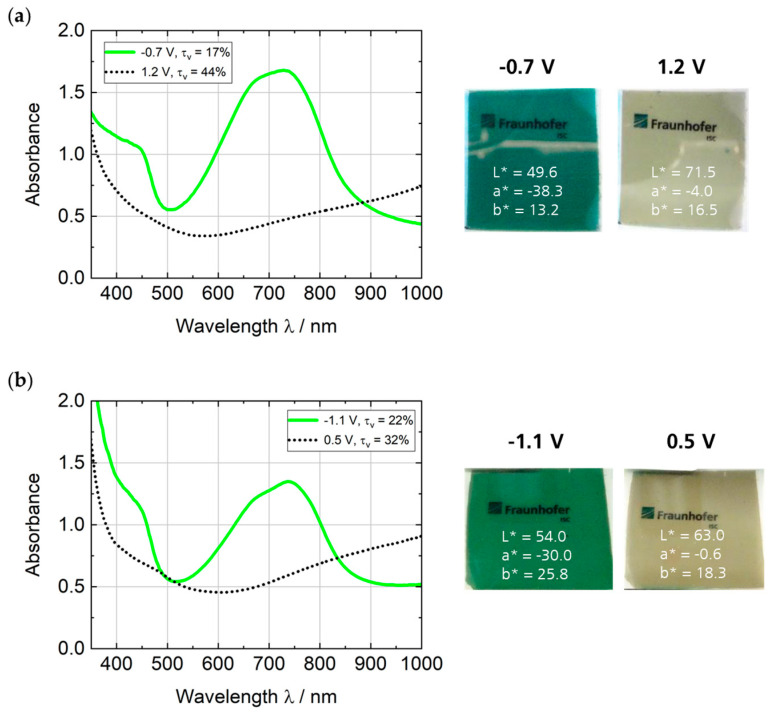
Absorption spectra, photographic images, and L*a*b* color coordinates of the hybrid ECDs (**a**) PEDOTPQ/PB and (**b**) PEDOTPQ/TiVO_x_ in the green-colored and bleached states, respectively. Electrolyte: LiTFSI/PC/DEC/paraloid.

PEDOTPQ as the cathodic EC material was tested in two different device configurations: (1) with PB as the complementarily coloring electrode (PEDOTPQ/PB) and (2) with TiVO_x_ as the optically passive ion storage electrode (PEDOTPQ/TiVO_x_). For the spectroelectrochemical characterization of these organic–inorganic (hybrid) ECDs, the cell voltage was increased in 0.1 V steps to determine the ideal operating voltages, where the maximum absorbance/transmittance and color changes are reached. The absorption spectra, as well as the photographic images and L*a*b* color coordinates of the PEDOTPQ-based EC cells in the green-colored and bleached states, respectively, are shown in Figure 11. The most relevant EC properties of both ECDs are summarized in Table 2. In the absorption spectra of the green-colored states at negative cell voltages (−0.7 V for PEDOTPQ/PB and −1.1 V for PEDOTPQ/TiVO_x_), the broad absorption peaks at 728 nm and 737 nm disappear when switched to the bleached states at positive cell voltages (+1.2 V and +0.5 V), resulting in a light-yellow coloration. In comparison, the ECD PEDOTPQ/PB shows a green-blue tint in the colored state and a higher visible light transmittance change between the colored and the bleached state (17%/44%) than the PEDOTPQ/TiVO_x_ cell (22%/32%). The complementarily coloring PB electrode enhances the optical contrast (contrast ratio: 2.6) and, thus, the EC effect.

**Figure 12:**
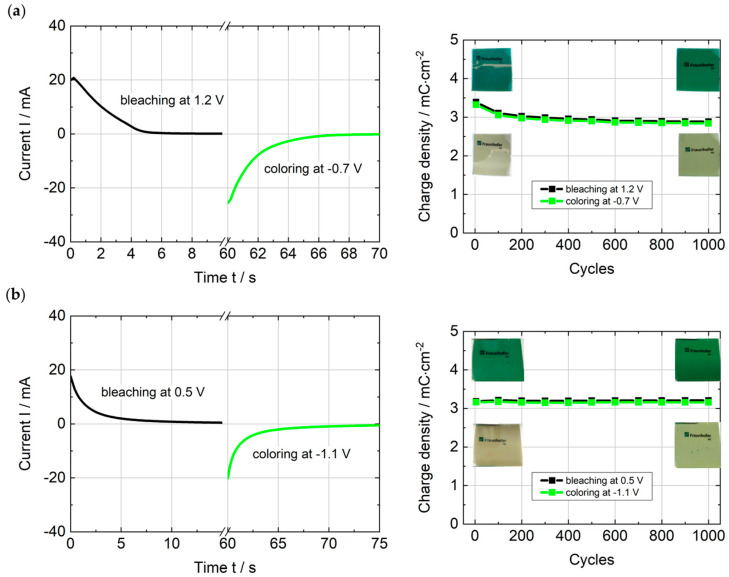
Current–time profile (**left**) and 1000 potentiostatic switching cycles (**right**) of the hybrid ECDs at ideal cell voltages: (**a**) PEDOTPQ/PB and (**b**) PEDOTPQ/TiVO_x_.

To assess the EC performance for applications, such as adaptive camouflage and displays, a key requirement in addition to the large optical contrast and distinct color change (i.e., high coloration efficiency) are short coloring and bleaching times [40], which were determined from the current–time profiles shown in Figure 12 (left). The switching time is defined as the time, after which the current drops to 10% of the initial value during the charging (bleaching) and discharging (coloring) process. Both hybrid ECDs exhibit a fast response of approx. 4 s (PEDOTPQ/PB) and 5 s (PEDOTPQ/TiVO_x_) for coloring and bleaching, respectively. The cycling stability of the ECDs was tested over 1000 potentiostatic cycles within the ideal voltage range for each cell configuration obtained from the spectroelectrochemical characterization (see Figure 11). The cell voltages at which the ECDs showed the maximum optical contrast were applied for 60 s. After each 100 cycles, the charge and discharge curves were measured at a current density of 50 μA·cm^−2^ to determine the charge density after bleaching and coloring (Figure A5).

**Figure A4:**
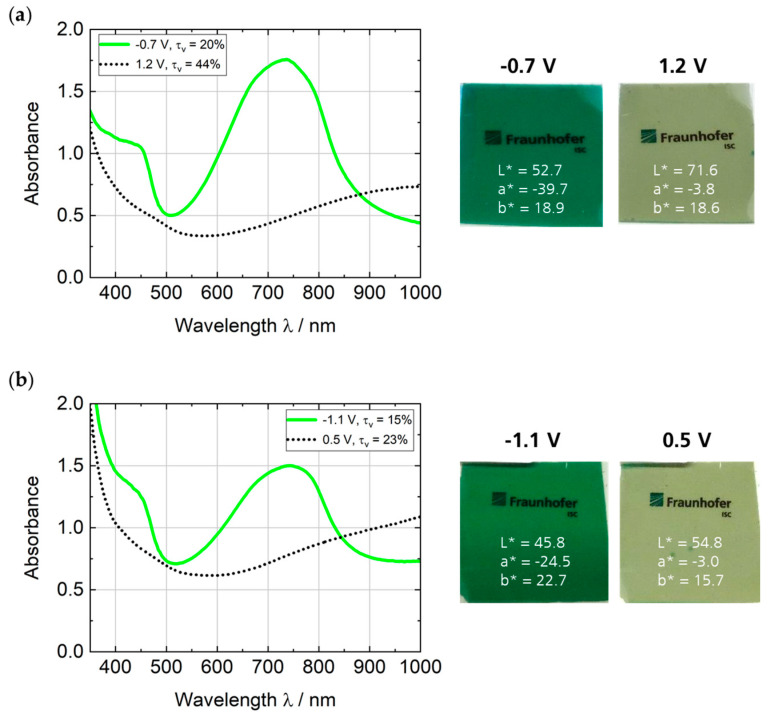
Absorption spectra, photographic images, and L*a*b* color coordinates of the hybrid ECDs (**a**) PEDOTPQ/PB and (**b**) PEDOTPQ/TiVO_x_ in the green-colored and bleached states, respectively, after 1000 switching cycles. Electrolyte: LiTFSI/PC/DEC/paraloid.

**Figure A5:**
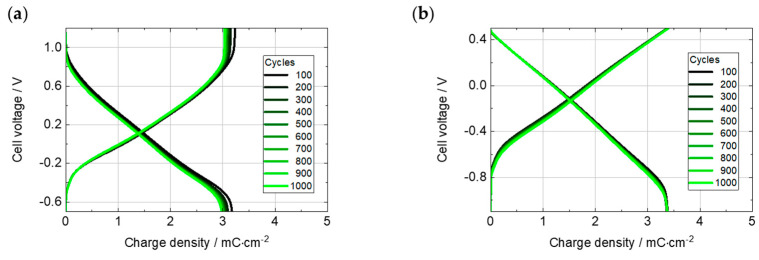
Charge and discharge curves (current density: 50 µA·cm^−1^) of the hybrid ECDs (**a**) PEDOTPQ/PB and (**b**) PEDOTPQ/TiVO_x_ over the course of 1000 switching cycles. Electrolyte: LiTFSI/PC/DEC/paraloid.

**Figure A6:**
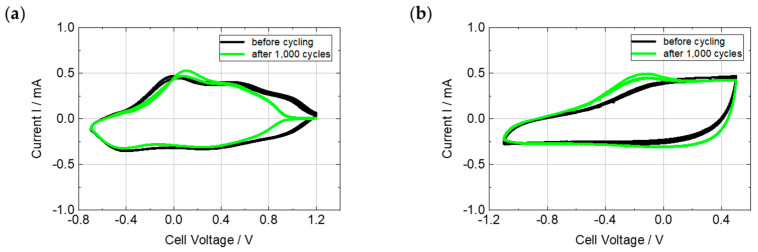
Cyclic voltammograms (scan rate: 10 mV·s^−1^) of the hybrid ECDs (**a**) PEDOTPQ/PB and (**b**) PEDOTPQ/TiVO_x_ before and after 1000 switching cycles. Electrolyte: LiTFSI/PC/DEC/paraloid.

In Figure 12a, it can be seen that the charge density of the PEDOTPQ/PB ECD decreases from 3.39 mC·cm^−2^ to 3.11 mC·cm^−2^ within the first 100 cycles. This loss of charge is presumably an indication of formation processes in the electrochemical cell during the first cycles, which can be explained by the slight charge imbalance of the PEDOTPQ and PB electrodes, as well as by hindered ionic diffusion in the electrolyte and insertion/extraction of ions into/from the EC layers during the accelerated cycling tests [18]. Afterwards, the charge density is more stable and reaches a value of 2.88 mC·cm^−2^ after 1000 cycles, which is close to the charge density of the PEDOTPQ electrode (2.94 mC·cm^−2^) determined in the half-cell measurement (see Figure 8). Therefore, an overall charge retention of 85% is achieved for the PEDOTPQ/PB ECD after 1000 switching cycles between −0.7 V and +1.2 V. In comparison, the PEDOTPQ/TiVO_x_ ECD (Figure 12b) shows no loss of charge over the course of 1000 switching cycles at −1.1 V/+0.5 V, resulting in a charge retention of 100% (3.16 mC·cm^−2^ → 3.16 mC·cm^−2^). The reason for the excellent cycling stability may be the high charge density of the TiVO_x_ electrode (>20 mC·cm^−2^) [37], which provides a large charge reservoir. Furthermore, the large charge excess of the ion storage layer (unbalanced configuration) has a positive effect on the operating cell voltages without compromising the optical contrast or switching time [41]. Finally, both hybrid ECDs show no significant change of the optical contrast or color in the bleached and colored states after 1000 potentiostatic switching cycles (see spectroelectrochemical measurements in Figure A4). The cyclic voltammograms of the cells before and after 1000 switching cycles confirm the high reversibility of the redox processes (Figure A6).

## 4. Conclusions and Outlook

The synthesis of a 5,8-bis(EDOT)quinoxaline monomer with two 4-(octyloxy)phenyl side chains (EDOTPQ) and the electropolymerization on ITO glass in a two-electrode setup with LiTf/PC/PEG-MEA as the electrolyte was successfully demonstrated in this study. The novel green polymer (PEDOTPQ) thin-film electrodes and devices exhibit interesting and promising EC properties for adaptive camouflage and display applications. Further potential applications are, e.g., smart labels or sensors. Two hybrid EC cell configurations with PEDOTPQ as the cathode and two different inorganic materials (PB and TiVO_x_) as complementarily coloring and minimally color-changing anodes, respectively, were assembled and tested to assess their functionality and EC performance. Both ECDs with an active area of 3.5 × 4 cm^2^ show a highly reversible and fast (<5 s) color change from green to light yellow. While the PEDOTPQ/PB cell has a higher visible light transmittance change (17%/44%) and a reasonable charge retention of 85% over 1000 potentiostatic switching cycles, the PEDOTPQ/TiVO_x_ cell (22%/32%) shows no loss of charge after 1000 cycles.

Further improvements addressing, e.g., the transmittance modulation and optical contrast by increasing the thickness of the EC polymer layer, as well as the long-term cyclability and durability of the hybrid EC cells, are needed to comply with the requirements of the aforementioned applications.

## Figures and Tables

**Table 1:** L*a*b* color coordinates, transmittance at λ_max_ in the colored (T_c_) and bleached states (T_b_), visible light transmittance (τ_v_), charge density (q), and coloration efficiency (η) of the EC electrodes.

	PEDOTPQ		PB	
	Colored	Bleached	Bleached	Colored
Potential vs. Li/Li^+^/V	2.4	3.5	2.4	3.4
L*	56.9	72.9	97.5	84.9
a*	−27.5	−2.1	−0.8	−13.0
b*	24.9	21.0	1.6	−13.3
λ_max_/%	736		677	
Transmittance at λ_max_/%	5	35	95	40
Visible light transmittance τ_v_/%	25	46	94	64
Max. charge density q/mC·cm^−2^	2.94	2.94	3.84	3.86
Coloration efficiency η at λ_max_/cm^2^·C^−1^	287		98	

**Table 2:** Characteristic EC properties of the hybrid ECDs: Cell voltage, L*a*b* color coordinates, transmittance at λ_max_ in the colored (T_c_) and bleached states (T_b_), visible light transmittance (τ_v_), contrast ratio, and coloring/bleaching time.

	PEDOTPQ vs. PB		PEDOTPQ vs. TiVO_x_	
	Colored	Bleached	Colored	Bleached
Cell voltage/V	−0.7	1.2	−1.1	0.5
L*	49.6	71.5	54.0	63.0
a*	−38.3	−4.0	−30.0	−0.6
b*	13.2	16.5	25.8	18.3
λ_max_/%	728		737	
Transmittance at λ_max_/%	2	34	4	26
Visible light transmittance τ_v_/%	17	44	22	32
Contrast ratio *	2.6		1.5	
Coloring/bleaching time/s	4		5	

* Contrast ratio = T_b_/T_c_.

**Figure A1:**
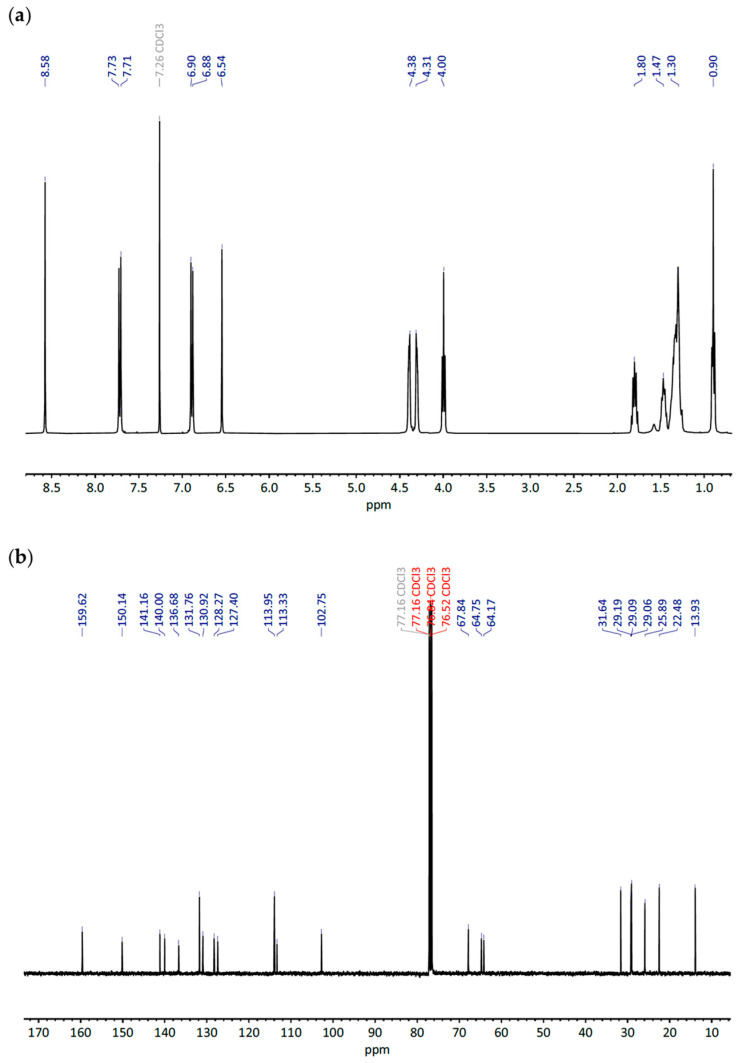
(**a**) ^1^H NMR and (**b**) ^13^C NMR spectra of the monomer EDOTPQ in CDCl_3_.

## Data Availability

Data are available from the corresponding author on reasonable request (due to privacy).
